# *Clostridium torrentis* sp. nov. and *Clostridium sajapyeongense* sp. nov., Anaerobic Bacteria Isolated from Soil in the Republic of Korea

**DOI:** 10.4014/jmb.2608.08027

**Published:** 2026-10-20

**Authors:** Yu Jeong Lee, Kira Moon, Byung Hee Chun

**Affiliations:** 1 Department of Microbiology, Pukyong National University, Busan 48513, Republic of Korea; 2 Department of Environmental Science, College of Natural Sciences, Hankuk University of Foreign Studies, Yongin 17035, Republic of Korea

**Keywords:** *Clostridium*, Novel species, Wetland, Soil, Anaerobic bacteria

## Abstract

Two obligately anaerobic bacterial strains, CS1-1^T^ and LS3^T^, were isolated from soil beside a waterfall and from alpine wetland soil, respectively, in Miryang, Republic of Korea, and characterized using a polyphasic taxonomic approach. Strains CS1-1^T^ and LS3^T^ showed the highest 16S rRNA gene sequence similarities of 98.69% and 98.75% to *Clostridium tanneri* A1-XYC3^T^ and *Clostridium tertium* DSM 2485^T^, respectively. Phylogenetic and phylogenomic analyses placed both strains within the genus *Clostridium* as distinct lineages. ANI values of strain CS1-1^T^ with *C. tanneri* A1-XYC3^T^ and of strain LS3^T^ with *C. tertium* DSM 2485^T^ were 79.3% and 83.7%, respectively, and the corresponding dDDH values were 25.1% and 27.8%, supporting their recognition as separate species. The genome sizes of strains CS1-1^T^ and LS3^T^ were 5.05 and 4.16 Mb, with G+C contents of 32.0% and 28.5%, respectively. Cells of both strains were Gram-stain-positive, flagellated, spore-forming rods and grew only under anaerobic conditions. The strains differed in phenotypic, physiological, biochemical, and chemotaxonomic characteristics. Strain LS3^T^ harbored a larger repertoire of carbohydrate-active enzyme genes than strain CS1-1^T^, consistent with its broader carbohydrate utilization in the API 20A system. Based on phylogenetic, genomic, phenotypic, and chemotaxonomic evidence, strains CS1-1^T^ and LS3^T^ represent two novel species, for which the names *Clostridium torrentis* sp. nov. and *Clostridium sajapyeongense* sp. nov. are proposed, respectively.

## Introduction

Wetlands are dynamic ecosystems characterized by high organic matter accumulation and frequent fluctuations in water saturation and redox conditions, creating diverse ecological niches for microorganisms [1]. Microbial communities in wetland soil play central roles in organic matter turnover by degrading complex carbohydrate-rich substrates and converting the resulting intermediates through anaerobic metabolic processes [2]. These processes involve the utilization of diverse carbohydrates and the production and transformation of organic acids, thereby contributing to carbon cycling [1, 3]. Such metabolic diversity among wetland microorganisms contributes to the efficient utilization of heterogeneous organic matter [4].

The genus *Clostridium* comprises a diverse group of Gram-stain-positive, spore-forming, anaerobic bacteria that are widely distributed in natural environments, including soil, sediments, and the gastrointestinal tracts of animals [5-11]. As of August 2026, the genus *Clostridium* comprises 172 species with validly published and correct names according to the List of Prokaryotic names with Standing in Nomenclature (LPSN) (https://lpsn.dsmz.de/genus/clostridium). Members of this genus exhibit considerable physiological and metabolic diversity and participate in the degradation and transformation of various organic compounds under anaerobic conditions [12, 13]. The genus is also characterized by substantial phylogenetic and phenotypic heterogeneity, which has prompted extensive taxonomic revisions and the reclassification of numerous species into newly established genera to improve its taxonomic coherence [14, 15]. Owing to this diversity and taxonomic complexity, accurate characterization of newly isolated *Clostridium* strains requires an integrated approach combining phylogenetic, genomic, phenotypic, and chemotaxonomic analyses [12, 16].

In this study, two novel anaerobic strains, CS1-1^T^ and LS3^T^, were isolated from soil beside Cheungcheung Waterfall and from wetland soil of the Sajapyeong alpine wetland, respectively, in Miryang, Republic of Korea. Analysis of 16S rRNA gene sequences indicated that both strains belonged to the genus *Clostridium* but were phylogenetically distinct from their closest relatives. Therefore, this study aimed to determine the taxonomic positions of strains CS1-1^T^ and LS3^T^ using a polyphasic taxonomic approach incorporating genome-based analyses, physiological and biochemical characterization, and chemotaxonomic analyses. Based on these results, strains CS1-1^T^ and LS3^T^ are proposed to represent two novel species of the genus *Clostridium*, for which the names *Clostridium torrentis* sp. nov. and *Clostridium sajapyeongense* sp. nov. are proposed, respectively.

## Materials and Methods

### Isolation of Strains

Strains CS1-1^T^ and LS3^T^ were isolated from soil collected at a depth of 15 cm beside Cheungcheung Waterfall (35°32′00.2″N, 128°59′09.1″E), Republic of Korea, and from wetland soil at Sajapyeong alpine wetland (35°32′04.0″N, 128°59′10.0″E), Republic of Korea, respectively. All subsequent procedures were performed in an anaerobic chamber (COY Laboratory Products, USA) containing an atmosphere of 90% N_2_, 5% CO_2_, and 5% H_2_. Soil samples were serially diluted using sterile phosphate-buffered saline, plated onto Reasoner’s 2A agar (R2A; MBcell, Republic of Korea) supplemented with 0.03% (w/v) sodium pyruvate, and incubated anaerobically at 30°C for 7 days. After incubation, colonies grown on R2A agar were randomly selected, and their 16S rRNA genes were amplified by PCR using the universal bacterial primers 27F and 1492R [17]. The PCR amplicons were sequenced using primers 337F (5′-GACTCCTACGGGAGGCWGCAG-3′), 785F (5′-GGATTAGATACCCTGGTA-3′), and 800R (5′-TACCAGGGTATCTAATCC-3′) by Macrogen (Republic of Korea). The nearly complete 16S rRNA gene sequences of strains CS1-1^T^ and LS3^T^ were assembled using BioEdit version 7.7.1 [18]. The obtained 16S rRNA gene sequences were compared with those of all type strains of species with validly published names using the EzBioCloud database [19]. Among the isolates, strains CS1-1^T^ and LS3^T^ were selected as candidates for novel species because they showed less than 99.0% 16S rRNA gene sequence similarity to their closest type strains. Strains CS1-1^T^ and LS3^T^ were preserved at -80°C in a single solution containing both 15% (v/v) glycerol and 15% (w/v) skim milk.

### Phylogenetic Analysis Based on 16S rRNA Gene Sequences

The nearly complete 16S rRNA gene sequences of strains CS1-1^T^ and LS3^T^ and those of closely related type strains of *Clostridium sensu stricto* [12] were aligned using the SILVA Incremental Aligner (SINA) version 1.2.12 [20]. Phylogenetic trees were reconstructed using the neighbor-joining (NJ), maximum-likelihood (ML), and maximum-parsimony (MP) algorithms implemented in MEGA11 [21]. The NJ, ML and MP trees were reconstructed using the Kimura two-parameter model, the Tamura-Nei model and the subtree-pruning-regrafting search, respectively, based on an alignment of 1,475 nucleotide positions. The robustness of the tree topologies was evaluated by bootstrap analysis based on 1,000 replicates.

### Whole-Genome Sequencing, Phylogenomic Analysis, and Functional Genomic Analysis

Genomic DNA of strains CS1-1^T^ and LS3^T^ was extracted from cells cultured in PY+X medium (peptone–yeast extract medium; KCTC Medium No. 778; per liter: 5.0 g Trypticase peptone, 5.0 g peptic digest of meat, 10.0 g yeast extract, 5.0 g D-glucose, 0.5 g L-cysteine-HCl·H_2_O, 40 mL salt solution and 0.5 ml Na-Resazurin solution (0.1%, w/v)) for strain CS1-1^T^ and Reinforced Clostridial Medium (RCM; MBcell) for strain LS3^T^ using the phenol-chloroform extraction method [22]. The genomic DNA of strains CS1-1^T^ and LS3^T^ was sequenced using the PacBio Revio (Phyzen, Republic of Korea). The sequenced reads were assembled *de novo* using Flye version 2.9.6 [23]. The quality of assembled genomes was evaluated based on their completeness and contamination using CheckM version 1.2.4 [24]. The complete genome sequences of strains CS1-1^T^ and LS3^T^ were annotated using the NCBI Prokaryotic Genome Annotation Pipeline (PGAP) version 6.4 and visualized using Proksee [25, 26].

Phylogenomic relationships of strains CS1-1^T^ and LS3^T^ with their closely related taxa of *Clostridium sensu stricto* [12] were analyzed using the Updated Bacterial Core Gene pipeline version 2 (UBCG2), based on the concatenated alignment of nucleotide sequences from 81 bacterial core genes from each genome [27]. An ML tree was reconstructed using MEGA11 [21] with the Tamura-Nei model based on a concatenated alignment of 69,174 nucleotide positions. To assess genome relatedness, average nucleotide identity (ANI) and digital DNA-DNA hybridization (dDDH) values between strains CS1-1^T^ and LS3^T^ and their closest type strains were calculated using OrthoANI and Genome-to-Genome Distance Calculator (GGDC; formula 2), respectively [28, 29].

Carbohydrate-active enzymes (CAZyme) were identified using the dbCAN3 meta server with the CAZy database [30]. KEGG Orthology identifiers were classified according to the KEGG BRITE hierarchy into 7 major functional categories and 12 second-level categories within Metabolism [31].

### Phenotypic, Physiological, and Biochemical Characterization

The optimal growth media of strains CS1-1^T^ and LS3^T^ were determined using R2A agar supplemented with 0.03% (w/v) sodium pyruvate, PY+X, Reinforced Clostridial Medium agar (RCM; MBcell, Republic of Korea), tryptic soy agar (TSA; MBcell, Republic of Korea), Brain Heart Infusion agar (BHI; MBcell, Republic of Korea), and Luria-Bertani agar (LB; MBcell, Republic of Korea). Growth at different temperatures was evaluated on the respective optimal agar media at 4–55°C. NaCl tolerance was assessed in the respective optimal broth media supplemented with 0–10% (w/v, with 1% interval) NaCl. Growth at different pH values was examined in the optimal broth media over a pH range of 4.5–9.5 at intervals of 0.5 pH units. The pH was adjusted using citrate (pH 4.5–5.5), Na_2_HPO_4_/NaH_2_PO_4_ (pH 6.0–8.5) and Tris/HCl (pH 9.0–9.5) buffers, and, when necessary, readjusted after autoclaving [32]. Aerobic growth was assessed under the respective optimal growth conditions. Unless otherwise specified, all growth tests were performed for 7 days under anaerobic conditions.

Cell morphology was examined using transmission electron microscopy (HT7800; Hitachi, Japan) after negative staining with 0.1% (w/v) phosphotungstic acid on formvar-coated copper grids. Gram staining was performed as described by Gram (1884), and endospore formation was examined using the Schaeffer-Fulton staining method [33, 34]. Oxidase and catalase activities were determined using an oxidase reagent (bioMérieux) and 3% hydrogen peroxide (Junsei, Japan), respectively. Hydrolytic activities were evaluated on their optimal growth agar media supplemented with 6% (w/v) starch and skim milk, and 6% (v/v) Tween 20 and Tween 80, as previously described [35].

Substrate utilization by strains CS1-1^T^ and LS3^T^ was assessed using the API 20A kit (bioMérieux) according to the manufacturer’s instructions, with all procedures performed under anaerobic conditions. In addition, fermentation end-products in culture supernatants collected after 2 days of incubation under the respective optimal growth conditions were analyzed using a 600 MHz Fourier-transform nuclear magnetic resonance spectrometer (FT-NMR; JNM ECA-600, JEOL, Japan) and the metabolites were identified and quantified using Chenomx version 12.0 (Chenomx Inc., Canada).

### Chemotaxonomic Characterization

For chemotaxonomic analyses, strains CS1-1^T^ and LS3^T^ were cultivated under their respective optimal growth conditions (PY+X for strain CS1-1^T^ and RCM for strain LS3^T^), as no common medium supported sufficient growth of both strains. Cells were harvested during the exponential growth phase, and cellular fatty acids were extracted according to the standard MIDI protocol of the Sherlock Microbial Identification System (version 6.2B). The extracted fatty acids were analyzed using a gas chromatograph (GC; Hewlett-Packard 6890, USA) and identified by comparison with the TSBA6 database [36].

Polar lipids were extracted using a chloroform-methanol method and separated by two-dimensional thin-layer chromatography (TLC) as described by Minnikin *et al*. (1977) [37], and individual lipids were tentatively identified by comparison of their positions and staining reactions with published reference profiles. Polar lipid profiles were visualized using 10% ethanolic molybdophosphoric acid for total polar lipids, ninhydrin for aminolipids, Dittmer-Lester reagent for phospholipids, and α-naphthol and sulfuric acid for glycolipids.

## Results and Discussion

### Phylogenetic Analysis Based on 16S rRNA Gene Sequences

Strains CS1-1^T^ and LS3^T^ showed the highest 16S rRNA gene sequence similarities of 98.69% and 98.75% to *Clostridium tanneri* A1-XYC3^T^ and *Clostridium tertium* DSM 2485^T^, respectively (Table S1). Phylogenetic analysis based on 16S rRNA gene sequences using the NJ algorithm showed that strains CS1-1^T^ and LS3^T^ each formed distinct lineage within the genus *Clostridium* (Fig. 1). Phylogenetic trees reconstructed using the ML and MP algorithms exhibited similar topologies (Fig. S1). Strain CS1-1^T^ clustered with *C. tanneri* A1-XYC3^T^ [11], a recently described member of *Clostridium sensu stricto*, whereas strain LS3^T^ clustered within the clade containing the type species *C. butyricum*. These results indicate that both strains belong to *Clostridium sensu stricto* (cluster I). However, because the 16S rRNA gene sequence similarity between strain LS3^T^ and *C. tertium* DSM 2485^T^ slightly exceeded the commonly applied species delineation threshold (98.70%), genome-based analyses were further conducted to clarify the taxonomic status [38].

### Genome-Based Taxonomic and Phylogenomic Analyses

The complete genome sequences of strains CS1-1^T^ and LS3^T^ were obtained by whole-genome sequencing and *de novo* assembly, revealing a single chromosome in strain CS1-1^T^ and one chromosome and two plasmids in strain LS3^T^ (Table 1; Fig. S2). Genome quality assessment revealed completeness of 99.38% for both strains and contamination of 1.86% and 2.17% for strains CS1-1^T^ and LS3^T^, respectively. These values met the commonly accepted criteria for high-quality bacterial genomes (≥90% completeness and ≤10% contamination) [24]. The genome sizes of strains CS1-1^T^ and LS3^T^ were 5,052,164 and 4,155,555 bp, respectively, with corresponding G+C contents of 32.0% and 28.5%. The genomes of strains CS1-1^T^ and LS3^T^ harbored 4,810 and 4,073 genes, respectively, including 4,521 and 3,914 protein-coding genes, 131 and 90 tRNA genes, 69 and 30 rRNA genes, and 13 and 3 ncRNA genes, respectively. The 69 rRNA genes of strain CS1-1^T^ correspond to 23 complete rRNA operons, an unusually high copy number; the 23 16S rRNA gene copies comprised 15 sequence variants (99.1–100% identity), and the PCR-derived sequence (PQ517338) was identical to the most frequent variant except for a single base-calling error near the 5′ end. The PCR-derived sequence of strain LS3^T^ (PX765903) was identical to 8 of the 10 genomic copies except for a single base-calling error near the 3′ end.

An ML phylogenomic tree reconstructed from the concatenated nucleotide sequences of 81 bacterial core genes exhibited a topology generally consistent with that of the 16S rRNA gene-based phylogenetic tree, with strains CS1-1^T^ and LS3^T^ occupying clearly distinct phylogenetic positions within the *Clostridium sensu stricto* (Fig. 2). Strain CS1-1^T^ clustered with *C. tanneri* A1-XYC3^T^, and strain LS3^T^ clustered within the clade containing the type species *C. butyricum*, supporting the placement of both strains in *Clostridium sensu stricto* (cluster I). In particular, strain LS3^T^ formed a distinct phylogenomic lineage from *C. tertium* DSM 2485^T^ despite their relatively high 16S rRNA gene sequence similarity. The ANI values of strain CS1-1^T^ with *C. tanneri* A1-XYC3^T^ and of strain LS3^T^ with *C. tertium* DSM 2485^T^ were 79.3% and 83.7%, respectively, and the corresponding dDDH values were 25.1% and 27.8%. These values were well below the species delineation threshold of 95–96% for ANI and 70% for dDDH [28, 29]. Collectively, these phylogenomic and genomic relatedness results support the classification of strains CS1-1^T^ and LS3^T^ as novel species within the genus *Clostridium*.

CAZyme analysis using the dbCAN3 meta server with the CAZy database predicted that the genomes of strains CS1-1^T^ and LS3^T^ encoded 81 and 122 CAZyme genes, respectively [30]. Despite having a larger genome and a greater number of protein-coding genes than strain LS3^T^, strain CS1-1^T^ harbored a smaller CAZyme repertoire (Table 1). This difference was primarily attributable to glycoside hydrolases (GHs), of which 21 and 60 genes were identified in strains CS1-1^T^ and LS3^T^, respectively. Accordingly, GHs represented 25.9% of the total CAZyme repertoire in strain CS1-1^T^ and 49.2% in strain LS3^T^, whereas the numbers of glycosyltransferases, carbohydrate esterases, and carbohydrate-binding modules were broadly comparable between the two strains. Differences in overall GH abundance were accompanied by distinct distributions of individual GH families (Fig. 3). Strain LS3^T^ possessed higher copy numbers of GH1 and contained multiple copies of GH2 and GH13, which were absent or less represented in strain CS1-1^T^, whereas GH18 and GH125 were more abundant in strain CS1-1^T^. These differences indicate distinct family-level CAZyme repertoires and suggest differences in the genetic potential for carbohydrate processing between the two strains. To further investigate this difference, the relative distribution of genes across KEGG functional categories was analyzed. At the first level of the KEGG hierarchy, metabolism accounted for the largest proportion of KEGG-assigned genes in both strains (Fig. 4A). However, the two strains exhibited distinct relative distributions among the second-level metabolic categories (Fig. 4B). Compared with strain LS3^T^, strain CS1-1^T^ showed a lower relative abundance of genes associated with carbohydrate metabolism, but higher relative abundances of genes related to amino acid metabolism and the metabolism of cofactors and vitamins. This pattern was broadly consistent with the relatively limited CAZyme repertoire of strain CS1-1^T^. Further analysis of carbohydrate metabolism revealed differences in the distribution of genes among individual pathways (Table S2). Strain LS3^T^ contained more genes associated with starch and sucrose metabolism and galactose metabolism, whereas strain CS1-1^T^ harbored more genes related to glyoxylate and dicarboxylate, propanoate, and butanoate metabolism.

### Phenotypic, Physiological, and Biochemical Characteristics

Strain CS1-1^T^ grew on R2A agar supplemented with 0.03% (w/v) sodium pyruvate and PY+X agar, with optimal growth observed on PY+X agar. In contrast, strain LS3^T^ grew on a wider range of media, including R2A agar supplemented with 0.03% (w/v) sodium pyruvate, RCM agar, TSA, and BHI agar, with optimal growth observed on RCM agar. Despite this difference in medium preference, the two strains exhibited generally similar growth conditions (Table 2). Strain CS1-1^T^ grew over a slightly broader temperature range than strain LS3^T^. Neither strain grew at pH 4.5 or 9.5, and both strains grew over broader pH ranges than their phylogenetically related taxa (Table 2), which may reflect the fluctuating conditions of their soil habitats [39-41]. Neither strain grew under aerobic conditions.

Cells of both strains were Gram-stain-positive rods, spore-forming and flagellated (Fig. S3). Cells of strain CS1-1^T^ measured 0.9–1.2 μm in width and 4.3–12.0 μm in length, whereas strain LS3^T^ measured 0.7–1.7 μm in width and 1.7–5.4 μm in length. Both strains were negative for catalase and oxidase activities and did not hydrolyze starch, casein, Tween 20, and Tween 80.

In the API 20A system, strain CS1-1^T^ hydrolyzed gelatin and esculin, whereas strain LS3^T^ hydrolyzed esculin and showed positive acidification reactions for D-glucose, D-lactose, D-maltose, salicin, cellobiose, mannose, and trehalose (Table S3). These phenotypic differences were consistent with the CAZyme analysis, which revealed that strain LS3^T^ possesses a larger CAZyme gene repertoire and a higher relative abundance of carbohydrate metabolism-related genes than CS1-1^T^, reflecting its broader carbohydrate utilization profile. In particular, the GH1 (β-glucosidase), GH2 (β-galactosidase) and GH13 (α-glucosidase) genes of strain LS3^T^ are consistent with its acid production from cellobiose, salicin, D-lactose, D-maltose and trehalose, whereas strain CS1-1^T^ did not produce acid from any of the carbohydrates tested. Neither strain hydrolyzed starch, indicating that the presence of GH13 genes does not necessarily translate into extracellular starch degradation under the tested conditions. Both strains produced butyrate and ethanol. In addition, strain CS1-1^T^ produced acetate and propionate, whereas strain LS3^T^ produced lactate. A detailed comparison of phenotypic, physiological, and biochemical characteristics is provided in Table 2 and the complete API 20A results are given in Table S3.

### Chemotaxonomic Characteristics

The major cellular fatty acids common to strains CS1-1^T^ and LS3^T^ were C_14:0_ and C_16:0_. In addition, summed feature 3 and C_18:1_ cis were identified as major fatty acids of strains CS1-1^T^ and LS3^T^, respectively (Table 3). As the two strains were grown in different media, these profiles are regarded as descriptive characteristics of each strain rather than as diagnostic differences between them. The two strains shared a similar polar lipid profile comprising diphosphatidylglycerol (DPG), aminophospholipid (APL), phospholipid (PL), glycolipid (GL), and unidentified lipid (UL). In total, strain CS1-1^T^ contained DPG, one APL, three PLs, one GL, and three ULs, whereas strain LS3^T^ contained DPG, two APLs, three PLs, two GLs, and four ULs (Figs. S4 and S5).

## Conclusion

Strains CS1-1^T^ and LS3^T^ were isolated from soil beside a waterfall and from alpine wetland soil, respectively, and represent two novel species within the genus *Clostridium*, as supported by 16S rRNA gene-based phylogenetic and phylogenomic analyses, together with ANI and dDDH analyses. Both strains were obligately anaerobic, Gram-stain-positive, flagellated, and spore-forming rods, but differed in several physiological, biochemical, and chemotaxonomic characteristics. Strain LS3^T^ possessed a larger CAZyme gene repertoire and a higher representation of carbohydrate metabolism-related genes, consistent with its broader carbohydrate utilization profile.

### Description of *Clostridium torrentis* sp. nov.

*Clostridium torrentis* (tor.ren’tis. L. gen. n. *torrentis*, referring to the waterfall environment from which the type strain was isolated)

Cells are Gram-stain-positive, flagellated rods, 0.9–1.2 μm in width and 4.3–12.0 μm in length, forming terminal endospores. Colonies on PY+X agar after 2 days under anaerobic conditions are small (<1 mm in diameter), whitish, irregular and flat. Growth occurs under anaerobic conditions, but not under aerobic conditions, at 20–40°C (optimum, 37°C), pH 5.0–9.0 (optimum, pH 7.0) and with 0–2% (w/v) NaCl (optimum, 0%). Grows on R2A agar supplemented with 0.03% (w/v) sodium pyruvate and on PY+X agar (optimum), but not on RCM, TSA, BHI or LB agar. Catalase and oxidase activities are negative. Starch, casein, Tween 20, and Tween 80 are not hydrolyzed. In the API 20A system, gelatin and esculin are hydrolyzed, but acid is not produced from any of the carbohydrates tested (D-glucose, D-mannitol, D-lactose, D-saccharose, D-maltose, salicin, D-xylose, L-arabinose, glycerol, cellobiose, mannose, melezitose, raffinose, sorbitol, rhamnose and trehalose). Butyrate and ethanol are produced as fermentation products, together with acetate and propionate. The major cellular fatty acids are C_14:0_, C_16:0_, and summed feature 3. The polar lipid profile contains DPG, APL, three PLs, GL, and three ULs.

The type strain, CS1-1^T^ (= KCTC 35034^T^ = JCM 38618^T^ = FBCC-B19276^T^), was isolated from soil collected beside Cheungcheung Waterfall in Miryang-si, Republic of Korea. The genome size is 5.05 Mb, with a G+C content of 32.0%. The GenBank accession numbers for the 16S rRNA gene and complete genome sequences are PQ517338 and CM148680, respectively. The genome assembly accession number is GCA_056135525.1.

### Description of *Clostridium sajapyeongense* sp. nov.

*Clostridium sajapyeongense* (sa.ja.pye.ong.en'se. N.L. neut. adj. *sajapyeongense*, pertaining to Sajapyeong, the locality from which the type strain was isolated)

Cells are Gram-stain-positive, flagellated rods, 0.7–1.7 μm in width and 1.7–5.4 μm in length, forming terminal endospores. Colonies on RCM agar after 2 days under anaerobic conditions are small (<1 mm in diameter), whitish, irregular and flat. Growth occurs under anaerobic conditions, but not under aerobic conditions, at 25–37°C (optimum, 37°C), pH 6.0–9.0 (optimum, pH 7.0) and with 0–2% (w/v) NaCl (optimum, 0%). Colonies grow on R2A agar supplemented with 0.03% (w/v) sodium pyruvate, RCM, TSA, and BHI agar, with optimal growth on RCM agar under tested conditions. Catalase and oxidase activities are negative. Starch, casein, Tween 20, and Tween 80 are not hydrolyzed. In the API 20A system, esculin is hydrolyzed, and positive acidification reactions are observed with D-glucose, D-lactose, D-maltose, salicin, cellobiose, mannose, and trehalose. Butyrate, ethanol, and lactate are produced as fermentation products. The major fatty acids are C_14:0_, C_16:0_, and C_18:1_ cis. The polar lipid profile contains DPG, two APLs, three PLs, two GLs, and four ULs.

The type strain, LS3^T^ (= KCTC 35090^T^ = JCM 38619^T^ = FBCC-B26315^T^), was isolated from wetland soil of the Sajapyeong alpine wetland in Miryang-si, Republic of Korea. The genome size is 4.16 Mb with a G+C content of 28.5%. The GenBank accession numbers for the 16S rRNA gene and complete genome sequences are PX765903 and CM143921, respectively, and those of the two plasmids are JBUZVV010000002 and JBUZVV010000003. The genome assembly accession number is GCA_055330225.1.

## Supplemental Materials

Supplementary data for this paper are available on-line only at http://jmb.or.kr.



## Figures and Tables

**Fig. 1:**
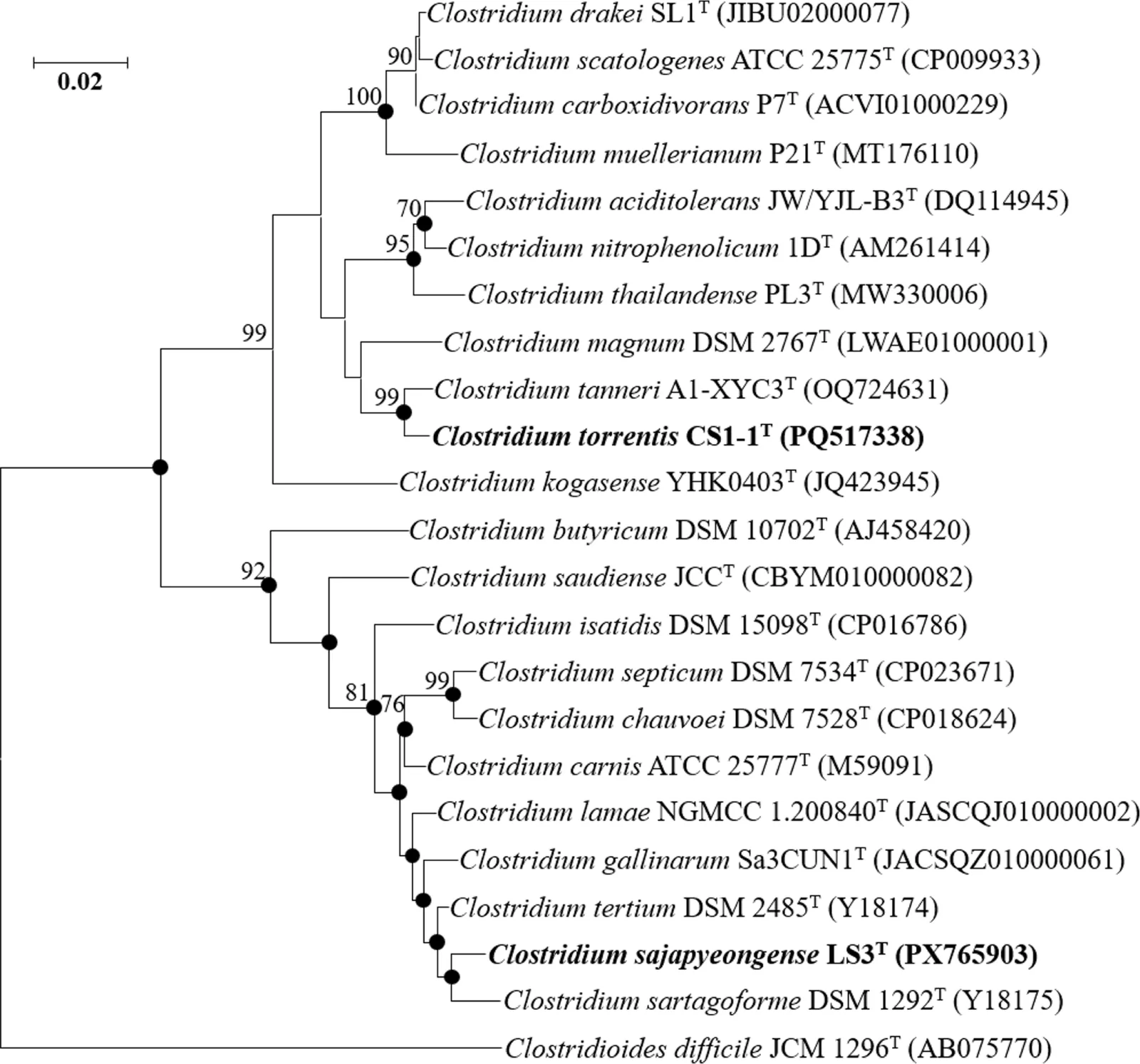
Neighbor-joining phylogenetic tree based on 16S rRNA gene sequences showing the relationships of strains CS1-1^T^ and LS3^T^ with members of *Clostridium sensu stricto*. Bootstrap values greater than 70% are shown at the nodes as percentages of 1,000 replicates. Filled circles ( ● ) indicate nodes that were also recovered in trees reconstructed using the maximum-likelihood and maximum-parsimony methods. *Clostridioides difficile* JCM 1296^T^ (AB075770) was used as the outgroup. The scale bar represents 0.02 substitutions per nucleotide position.

**Fig. 2:**
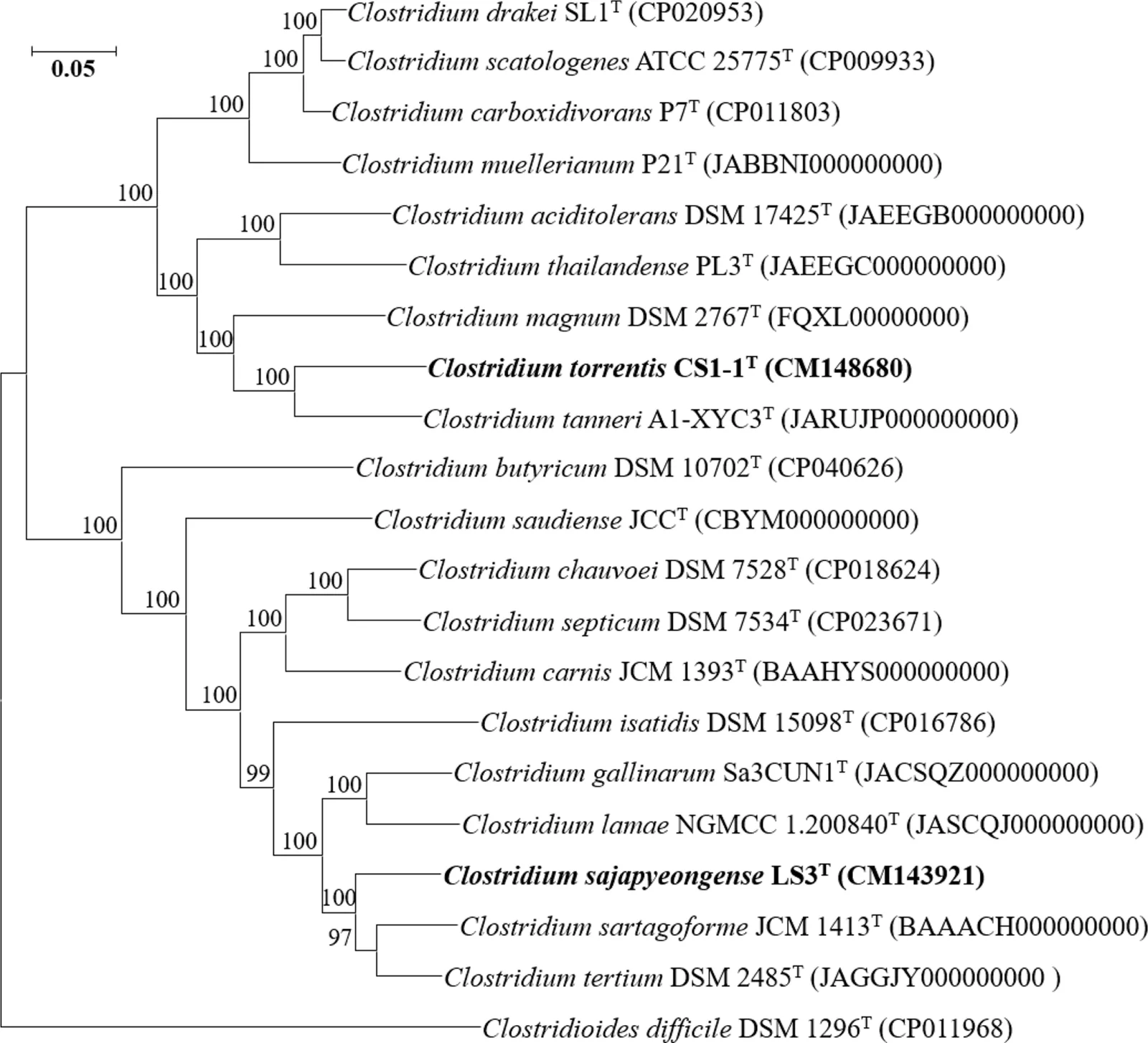
Maximum-likelihood phylogenomic tree showing the relationships of strains CS1-1^T^ and LS3^T^ with members of *Clostridium sensu stricto*, based on the concatenated sequences of 81 bacterial core genes. Bootstrap values greater than 70% are shown at the nodes as percentages of 1,000 replicates. *Clostridioides difficile* DSM 1296^T^ (CP011968) was used as the outgroup. The scale bar represents 0.05 substitutions per nucleotide position.

**Fig. 3:**
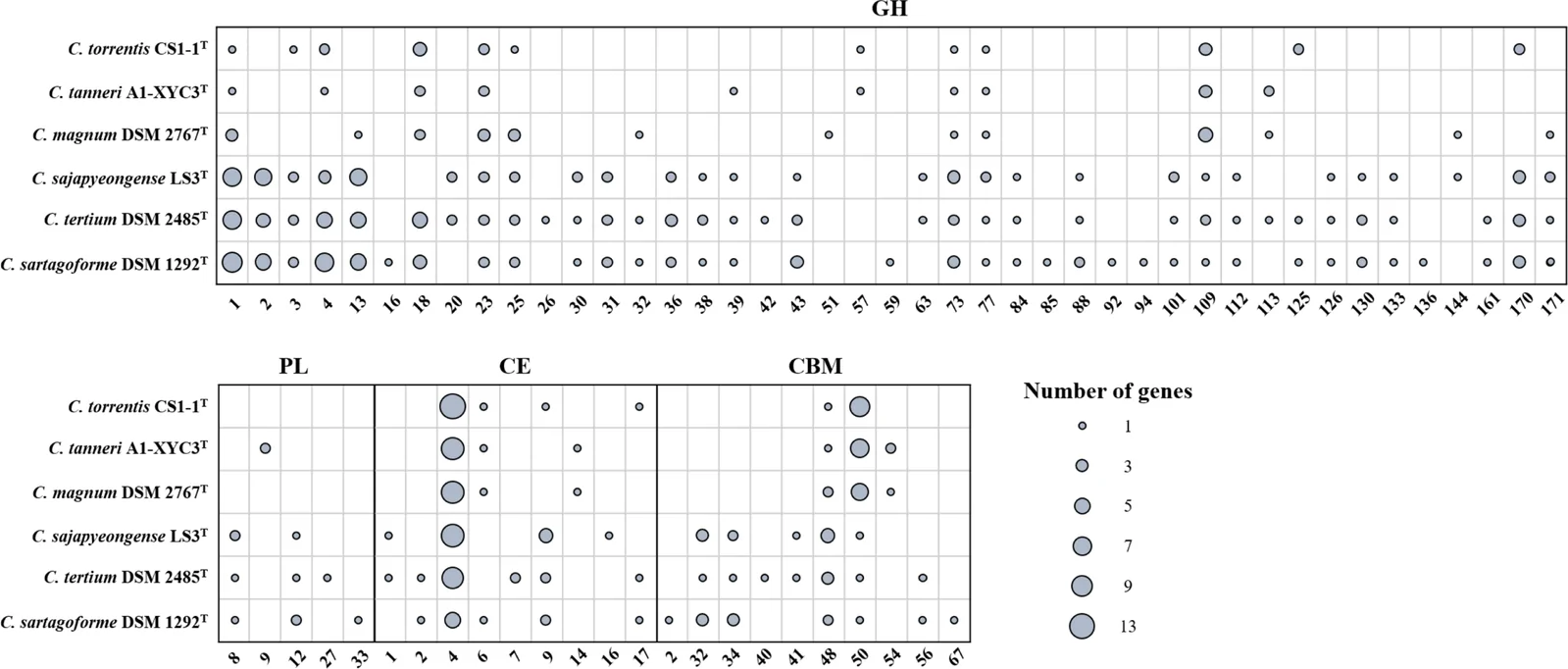
Distribution of carbohydrate-active enzyme (CAZyme) families in strains CS1-1^T^ and LS3^T^ and their closely related taxa. CAZyme genes were classified into glycoside hydrolase (GH), polysaccharide lyase (PL), carbohydrate esterase (CE), and carbohydrate-binding module (CBM) families. Bubble size represents the number of genes assigned to each CAZyme family.

**Fig. 4:**
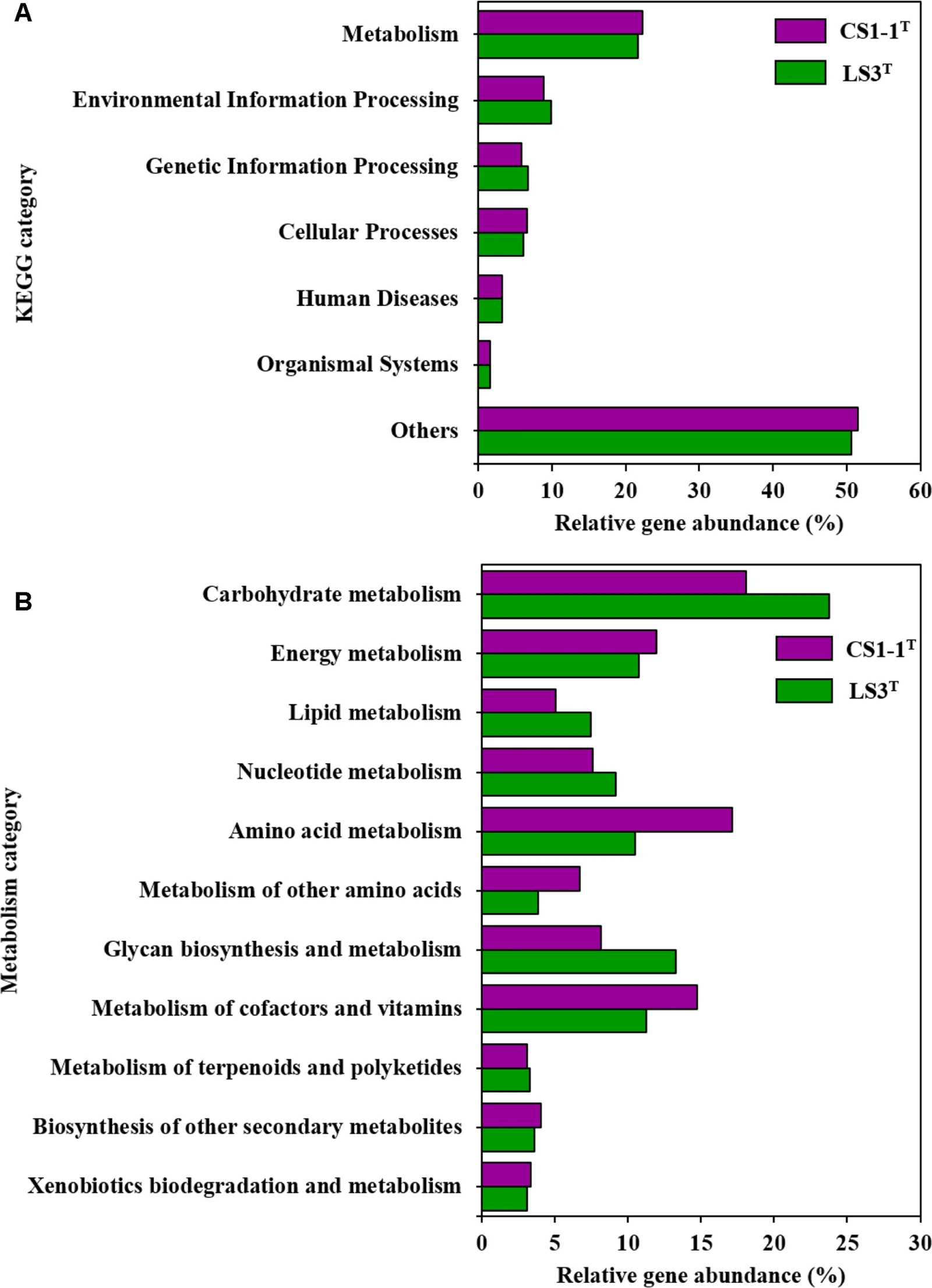
Functional classification of genes in strains CS1-1^T^ and LS3^T^ based on the KEGG BRITE hierarchy. Distribution of genes across seven major functional categories (A). Distribution of genes across 12 second-level functional categories within Metabolism (B). “Not included in regular maps” is omitted because neither strain had genes assigned to this category. Bars represent the relative abundance (%) of genes assigned to each functional category among KEGG-annotated genes.

**Table 1:** **Genome features of strains CS1-1^T^ and LS3^T^, and their closely related taxa.** Strains: 1, *C. torrentis* CS1-1^T^ (this study); 2, *C. tanneri* A1-XYC3^T^; 3, *C. magnum* DSM 2767^T^; 4, *C. sajapyeongense* LS3^T^ (this study); 5, *C. tertium* DSM 2485^T^; 6, *C. sartagoforme* DSM 1292^T^. NR, not reported.

	1	2	3	4	5	6
Genome size (Mb)	5.1	4.1	6.5	4.2	3.9	3.9
Status	Complete	Draft	Scaffold	Complete	Draft	Scaffold
G+C content (%)	32.0	32.5	32.0	28.5	28.0	28.0
N50	5.1 Mb	150.7 kb	123.4 kb	4 Mb	184.5 kb	182.3 kb
No. of total genes	4,810	3,829	6,252	4,073	3,733	3,726
No. of protein-coding genes	4,521	3,711	6,104	3,914	3,602	3,642
No. of total RNA genes	213	86	NR	123	92	NR
No. of tRNA genes	131	66	NR	90	76	NR
No. of rRNA genes	69	11	NR	30	13	9
No. of ncRNA genes	13	9	NR	3	3	NR
No. of pseudogenes	76	32	NR	36	39	NR
No. of total CAZyme genes	81	75	102	122	139	132
Glycoside hydrolase	21	15	23	60	69	73
Glycosyltransferase	33	35	56	30	39	31
Polysaccharide lyases	0	2	0	3	3	4
Carbohydrate esterase	16	12	12	16	16	10
Auxiliary activity	2	1	2	2	3	2
Carbohydrate-binding module	9	10	9	11	9	12
Coverage (×)	738	58	172	709	179	146
GenBank accession No.	CM148680	JARUJP000000000	FQXL00000000	CM143921	JAGGJY000000000	BAAACH000000000

**Table 2:** **Phenotypic characteristics of strains CS1-1^T^ and LS3^T^, and their closely related taxa.** Strains: 1, *C. torrentis* CS1-1^T^ (this study); 2, *C. tanneri* A1-XYC3^T^ [11]; 3, *C. magnum* DSM 2767^T^ [42]; 4, *C. sajapyeongense* LS3^T^ (this study); 5, *C. tertium* DSM 2485^T^ [43];6, *C. sartagoforme* DSM 1292^T^ [43]. A, acetate; B, butyrate; E, ethanol; F, formate; L, lactate; P, propionate. +, positive; −, negative; NR, not reported.

	1	2	3	4	5	6
Isolation source	Soil beside a waterfall	Alpaca feces	Enriched sediment	Wetland soil	Human wound	Garden soil
Gram-staining	+	–	+	+	+	+
Growth range of:						
Temperature (optimum, °C)	20−40 (37)	20−45 (37)	15−45 (30−32)	25−37 (37)	NR	NR
NaCl (optimum, %)	0.0−2.0 (0.0)	0.0−2.0 (0.5)	NR	0.0−2.0 (0.0)	NR	NR
pH (optimum)	5.0−9.0 (7.0)	6.0−7.0 (7.0)	6.0−7.5 (7.0)	6.0−9.0 (7.0)	NR	NR
Spore formation	+	–	+	+	+	+
End-products	A, B, E, P	A, E	A	B, E, L	A, B, L	A, B, F

**Table 3:** **Cellular fatty acid composition of strains CS1-1^T^ and LS3^T^, and their closely related taxa.** Strains: 1, *C. torrentis* CS1-1^T^ (this study); 2, *C. tanneri* A1-XYC3^T^ [11]; 3, *C. sajapyeongense* LS3^T^ (this study); 4, *C. tertium* DSM 2485^T^ [44]. The major fatty acids (> 5%) are highlighted in bold. tr, trace (< 1%); –, not detected.

	1	2	3	4
Saturated:				
C_11:0_	tr	-	-	-
C_12:0_	tr	1.0	2.5	**7.2**
C_13:0_	-	1.4	-	-
C_13:0_ iso	-	2.7	-	-
C_13:0_ anteiso	-	1.6	-	-
C_14:0_	**12.3**	**21.6**	**7.1**	**13.4**
C_14:0_ 3-OH	tr	-	-	-
C_15:0_	3.6	-	-	-
C_15:0_ anteiso	1.6	2.0	-	-
C_16:0_	**18.8**	**14.1**	**37.4**	**37.3**
C_16:0_ iso	tr	-	-	1.2
C_16:0_ 2-OH	tr	-	-	-
C_17:0_	-	1.3	-	-
Unsaturated:				
C_15:1_ ω6c	-	2.2	-	-
C_15:1_ ω8c	-	1.5	-	-
C_17:1_ ω8c	-	1.2	-	-
C_18:1_ cis	-	-	**11.5**	-
Summed Feature^a^				
3	**15.4**	**7.3**	4.0	-
4	-	**6.2**	-	-
5	-	4.1	-	-
10	2.6	**12.8**	3.7	**7.5**
12	1.6	**5.7**	-	tr

^a^Two or three fatty acids could not be separated by the Microbial Identification System. Summed feature 3 comprises C_16:1_ ω7c and/or C_16:1_ ω6c. Summed feature 4 comprises unknown C_15.040_/C_15.0_. Summed feature 5 comprises C_18:2_ ω6,9c/C_18:0_ anteiso. Summed feature 10 comprises C_18:0_ and/or C_17:0_ cyc. Summed feature 12 comprises unknown C_14.600_/C_15:0_ iso
