# Supplementary material for: Clostridium torrentis sp. nov. and Clostridium sajapyeongense sp. nov., Anaerobic Bacteria Isolated from Soil in the Republic of Korea
Source: J Microbiol Biotechnol. 2026 Oct 20;36:e2608027. doi: 10.4014/jmb.2608.08027 (PMC13754562; doi:10.4014/jmb.2608.08027)
Supplement: Supplementary file 1 [file jmb-36-e2608027-supple.pdf]

J. Microbiol. Biotechnol. 2026. 36: e2608027

https://doi.org/10.4014/jmb.2608.08027

Supplementary Tables and Figures

Clostridium torrentis sp. nov. and *Clostridium sajapyeongense* sp. nov., isolated from soil in Republic of Korea

Yu Jeong Lee[1], Kira Moon[2], and Byung Hee Chun[1*]

[1]*Department of Microbiology, Pukyong National University, Busan 48513, Republic of Korea*

[2]*Department of Environmental Science, College of Natural Sciences, Hankuk University of Foreign Studies, Yongin 17035, Republic of Korea*

Running title: *Clostridium torrentis* sp. nov. and *Clostridium sajapyeongense* sp. nov

* Corresponding author, bhchun@pknu.ac.kr (B. H. Chun).

Table S1. 16S rRNA gene sequence similarities between strain CS1-1^{T}, LS3^{T}, and their closely related type strains.

Strain	Closely related type strain	Accession No.	similarity (%)
CS1-1^{T}	*Clostridium tanneri* A1-XYC3^{T}	OQ724631	98.69
	Clostridium magnum DSM 2767^{T}	LWAE01000001	97.06
	Clostridium carboxidivorans P7^{T}	ACVI01000229	96.50
LS3^{T}	*Clostridium tertium* DSM 2485^{T}	Y18174	98.75
	Clostridium gallinarum Sa3CUN1^{T}	JACSQZ010000061	98.61
	Clostridium sartagoforme DSM 1292^{T}	Y18175	98.26

Table S2. Distribution of genes assigned to carbohydrate metabolism pathways in strains CS1-1^T and LS3^T. Values represent the number of genes assigned to each KEGG carbohydrate metabolism pathway.

Carbohydrate metabolism pathway	Numbers of genes:	
	CS1-1^T	LS3^T
Glycolysis / Gluconeogenesis	54	49
Citrate cycle (TCA cycle)	17	11
Pentose phosphate pathway	30	32
Pentose and glucuronate interconversions	10	10
Fructose and mannose metabolism	26	25
Galactose metabolism	14	41
Ascorbate and aldarate metabolism	2	9
Starch and sucrose metabolism	24	60
Inositol phosphate metabolism	9	3
Pyruvate metabolism	56	44
Glyoxylate and dicarboxylate metabolism	33	16
Propanoate metabolism	32	18
Butanoate metabolism	42	22
C5-Branched dibasic acid metabolism	8	0

Table S3. API 20A test results of strains CS1-1^T and LS3^T. +, positive; −, negative.

	CS1-1^T	LS3^T
Activities of API 20A:		
L-tryptophane	−	−
Urea	−	−
D-glucose	−	+
D-mannitol	−	−
D-lactose	−	+
D-saccharose	−	−
D-maltose	−	+
Salicin	−	+
D-xylose	−	−
L-arabinose	−	−
Gelatin	+	−
Esculin	+	+
Glycerol	−	−
Cellobiose	−	+
Mannose	−	+
Melezitose	−	−
Raffinose	−	−
Sorbitol	−	−
Rhamnose	−	−
Trehalose	−	+

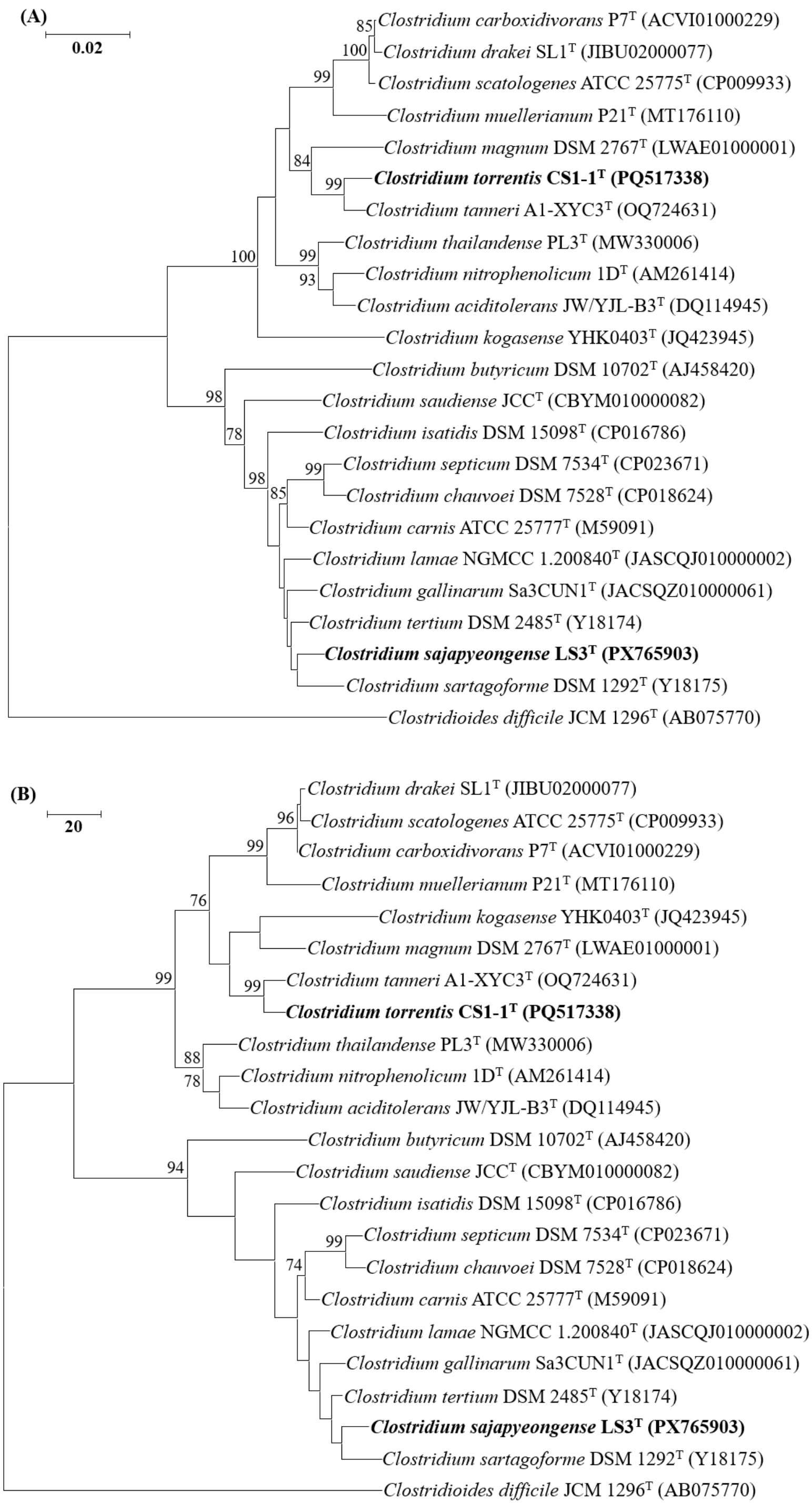

Fig. S1. Maximum-likelihood and maximum-parsimony phylogenetic trees based on 16S rRNA gene sequences showing the relationship of strains CS1-1^T and LS3^T with members of *Clostridium sensu stricto*. Bootstrap values with more than 70% are shown on the nodes as percentages of 1,000 replicates. *Clostridioides difficile* JCM 1296^T (AB075770) was selected as the outgroup.

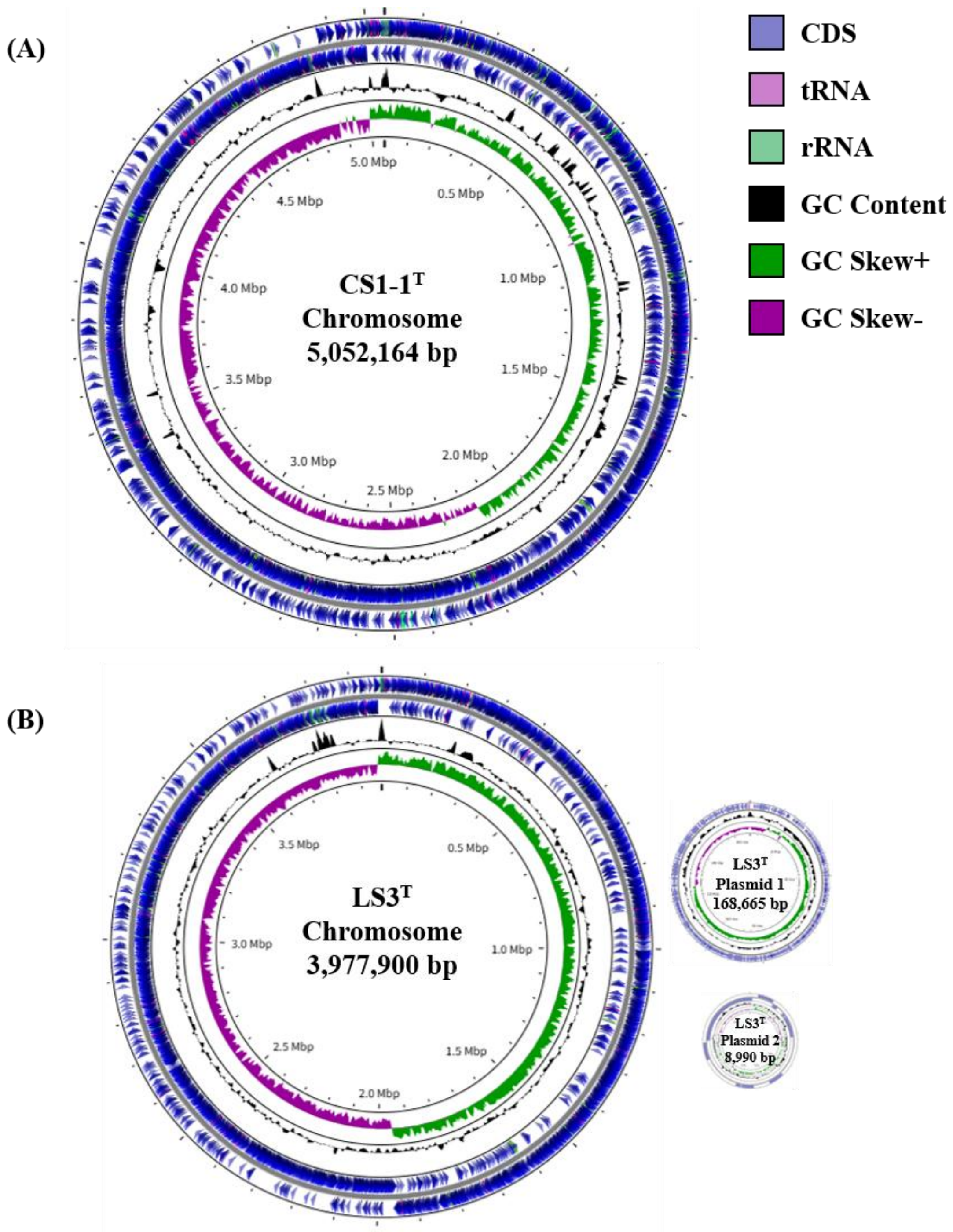

Fig. S2. Genome maps of strains CS1-1^{T} (A) and LS3^{T} (B). From outer to inner, tracks show CDSs on both strands, tRNA, rRNA, GC contents, and GC skew.

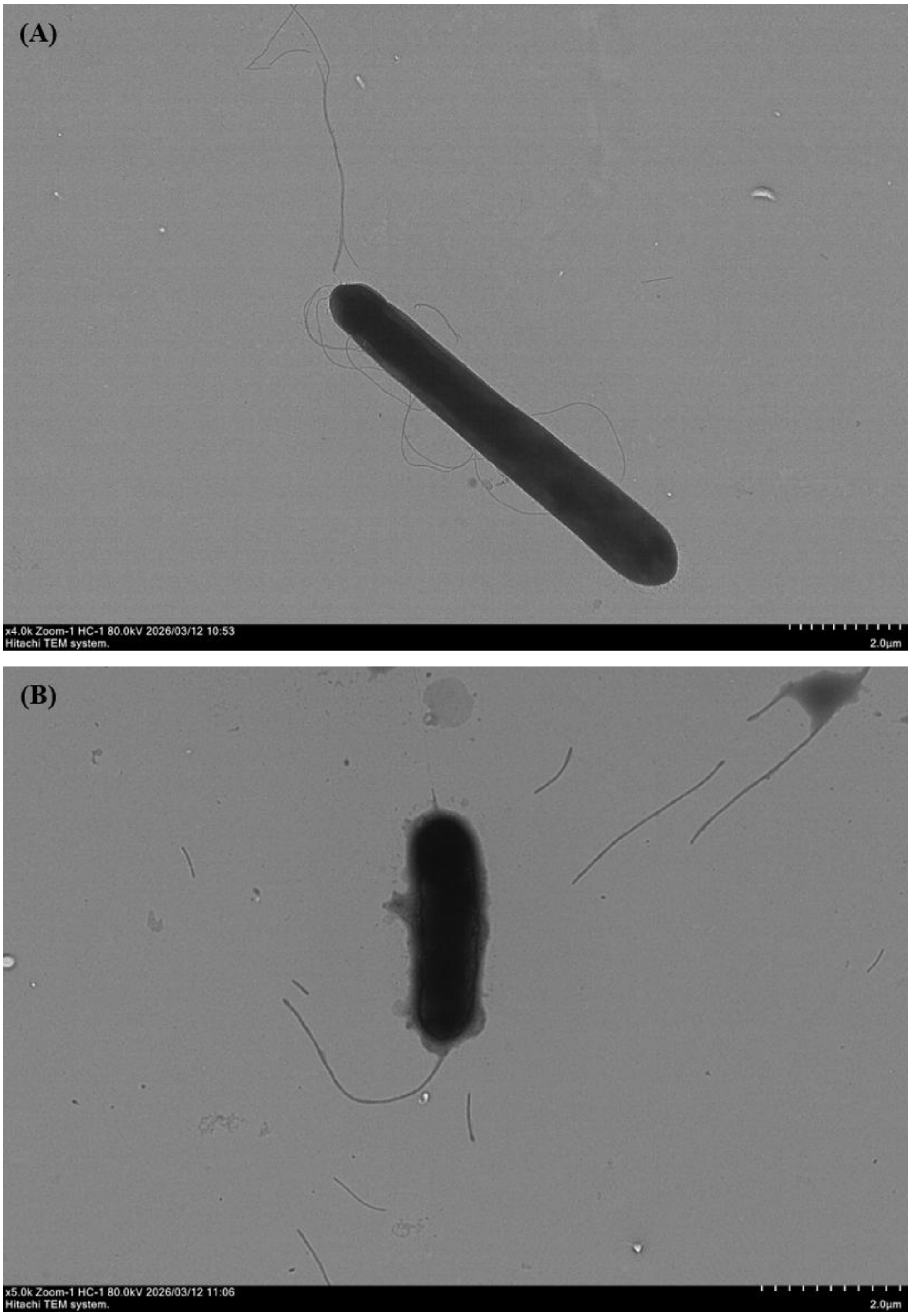

Fig. S3. Transmission electron micrograph of strains CS1-1^T (A) and LS3^T (B) cells. Bars, 2.0 µm.

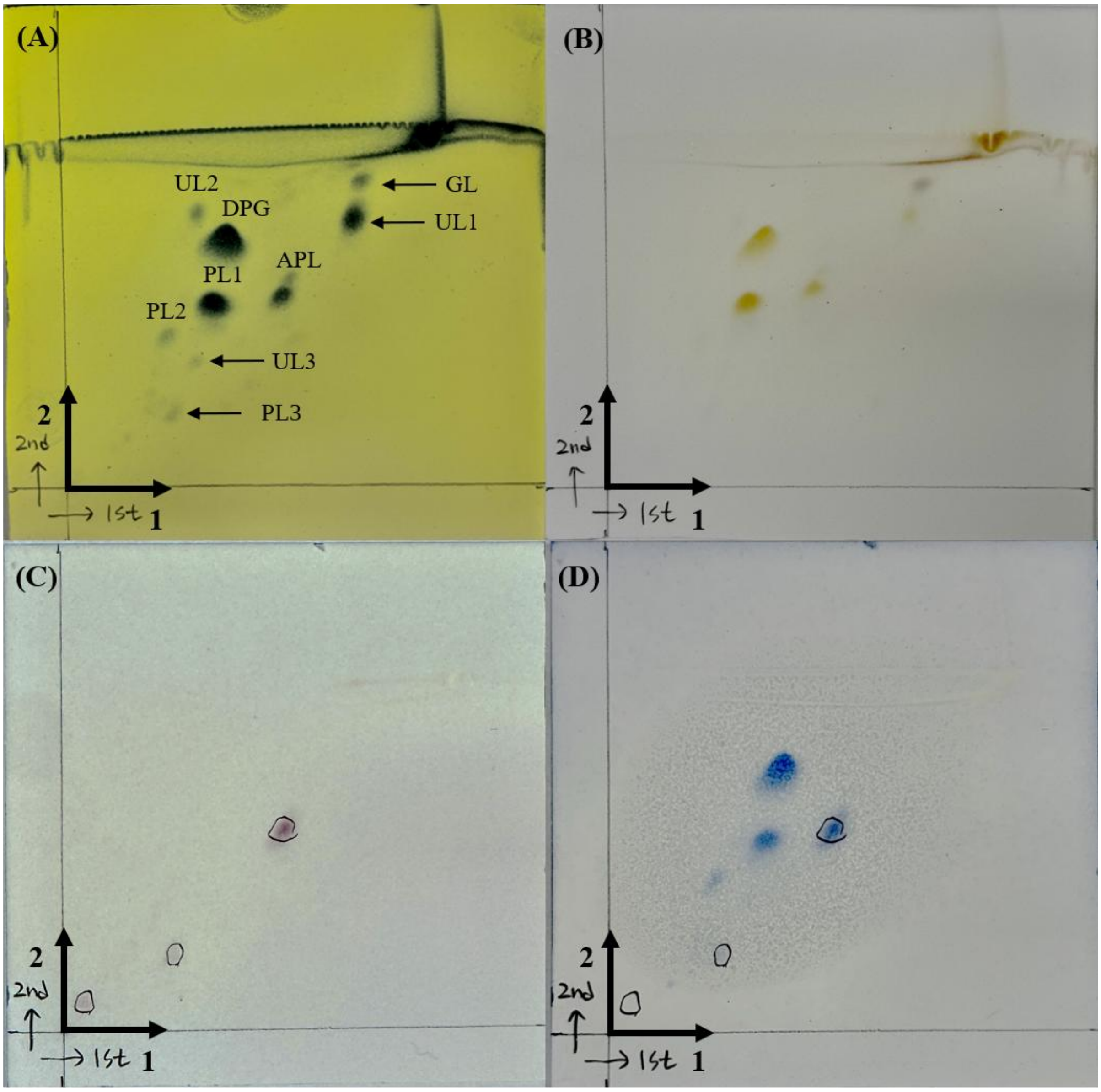

Fig. S4. Two-dimensional thin-layer chromatograms of the polar lipids of strain CS1-1^T, showing total lipids (A), glycolipids (B), aminolipids (C) and phospholipids (D). Total lipids were stained with 10% ethanolic molybdophosphoric acid, glycolipids with α-naphthol and sulfuric acid, aminolipids with ninhydrin, and phospholipids with ninhydrin followed by the Dittmer–Lester reagent. Solvent systems: first dimension, chloroform–methanol–water (65:25:4, v/v/v); second dimension, chloroform–acetic acid–methanol–water (80:15:12:4, v/v/v/v). DPG, diphosphatidylglycerol; APL, aminophospholipid; GL, glycolipid; PL, unidentified phospholipid; UL, unidentified polar lipid.

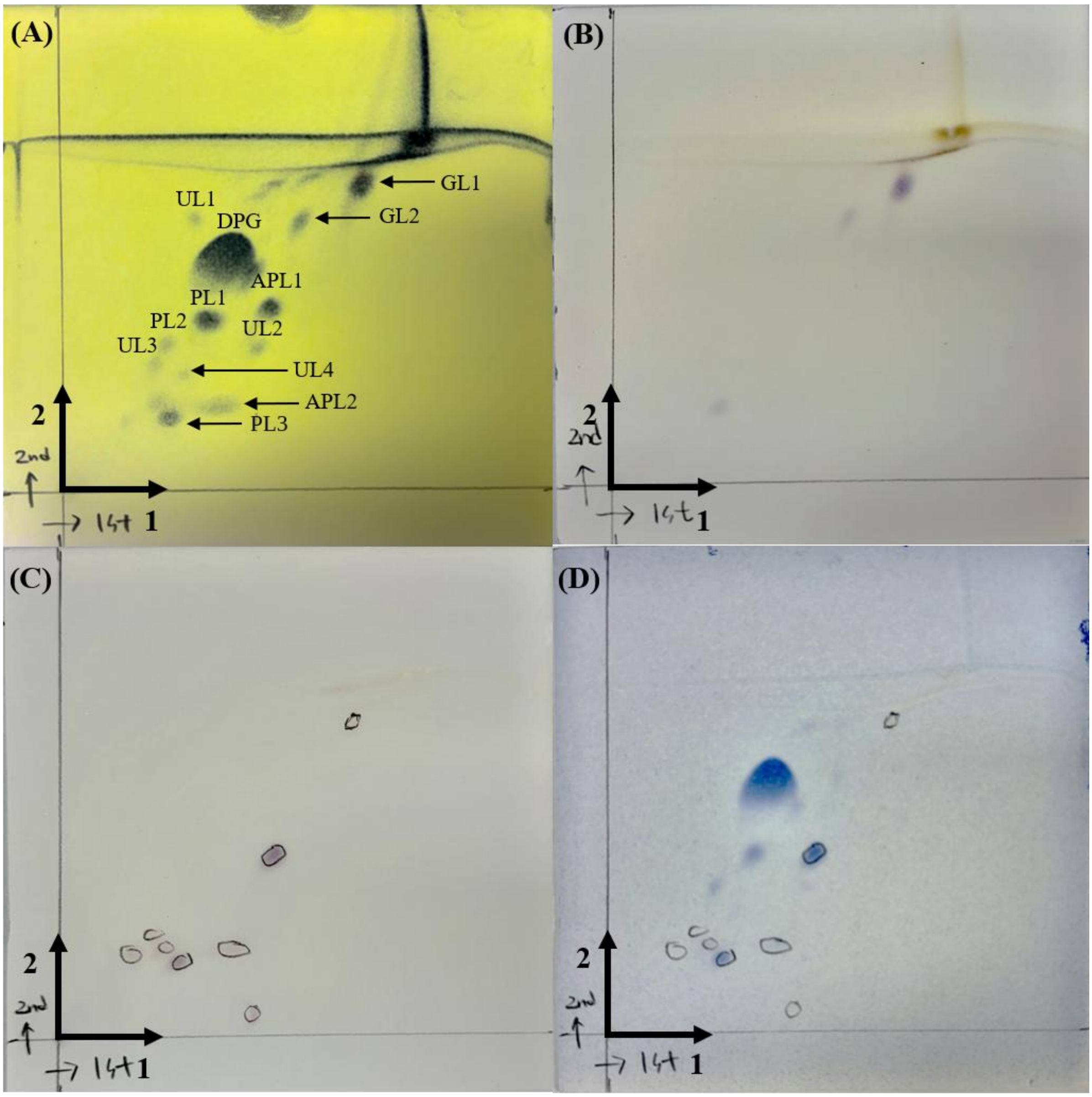

Fig. S5. Two-dimensional thin-layer chromatograms of the polar lipids of strain LS3^{T}, showing total lipids (A), glycolipids (B), aminolipids (C) and phospholipids (D). Staining and solvent systems were as described for Fig. S4. DPG, diphosphatidylglycerol; APL, aminophospholipid; GL, glycolipid; PL, unidentified phospholipid; UL, unidentified polar lipid.
